# Human-like smelling of a rose scent using an olfactory receptor nanodisc-based bioelectronic nose

**DOI:** 10.1038/s41598-018-32155-1

**Published:** 2018-09-17

**Authors:** Minju Lee, Heehong Yang, Daesan Kim, Myungjae Yang, Tai Hyun Park, Seunghun Hong

**Affiliations:** 1 Department of Physics and Astronomy and Institute of Applied Physics, Seoul National University, Seoul, 08826 Korea; 2 School of Chemical and Biological Engineering, Seoul National University, Seoul, 08826 Korea; 3 Protein Engineering Laboratory, Recombinants Unit, MOGAM Institute for Biomedical Research, Yongin, 16924 Korea; 4 Department of Biophysics and Chemical Biology, Seoul National University, Seoul, 08826 Korea

## Abstract

We report a strategy for the human-like smelling of a rose scent utilizing olfactory receptor nanodisc (ND)-based bioelectronic nose devices. In this strategy, a floating electrode (FE)-based carbon nanotube (CNT) field effect transistor (FET) was functionalized with human olfactory receptor 1A2 (hOR1A2)-embedded NDs (hOR1A2NDs). The hOR1A2NDs responded to rose scent molecules specifically, which were monitored electrically using the underlying CNT-FET. This strategy allowed us to quantitatively assess the contents of geraniol and citronellol, the main components of a rose scent, as low as 1 fM and 10 fM, respectively. In addition, it enabled us to selectively discriminate a specific rose odorant from other odorants. Significantly, we also demonstrated that the responses of hOR1A2NDs to a rose scent could be strongly enhanced by enhancer materials like a human nose. Furthermore, the method provided a means to quantitatively evaluate rose scent components in real samples such as rose oil. Since our method allows one to quantitatively evaluate general rose scent ingredients just like a human nose, it could be a powerful strategy for versatile basic research and various applications such as fragrance development.

## Introduction

A rose scent is known to be a pleasant smell to humans, and it has been used as a key component to impart scents to various fragrances and flavorings^1^. Some of the well-known ingredients for a rose scent are geraniol, citronellol, phenylethyl alcohol, nerol, and so on^2–4^. Rose oil products including such ingredients have often been utilized as one of the base materials for developing new perfumes, making the perfumes more complete and plentiful^5,6^. However, the overdose of such rose scent ingredients can cause bad smells and even allergic reactions to humans. Thus, the quantitative evaluation of rose scent ingredients in real samples such as rose oil can be an important issue and has been extensively studied in various areas such as cosmetic and pharmaceutical industries^7,8^. However, most of methods allow one to measure only specific well-known substances, and they cannot be used to measure how humans would respond to some new substances. Furthermore, they often exhibited a rather low selectivity compared with a human nose.

In humans and mammals, an olfactory system enables the discrimination of specific chemical components from other non-specific components, which has been critical in evaluating food quality and recognizing dangers in various environments^9,10^. In an olfactory system, olfactory receptor (OR) proteins recognize and bind only to specific odorant molecules, enabling the identification of specific smells^11^. For example, the main ingredients of a rose scent, geraniol and citronellol, could specifically bind to human olfactory receptor 1A2 (hOR1A2) with different characteristics^5,12,13^. Such selective binding characteristics of ORs have been utilized to develop bioelectronic nose sensors with human-like responses^11,14^. For example, versatile bioelectronic noses have been developed by hybridizing ORs onto electric channels based on various nanostructures such as carbon nanotubes (CNTs), conducting polymer nanotubes, and graphene^15–17^. However, it is often difficult to ensure the structural stability of ORs and to achieve the stable immobilization of ORs on such nanostructured material surfaces, degrading the stability and sensitivity of the bioelectronic nose devices based on them^15,18^. Furthermore, bioelectronic nose devices to detect general rose scent ingredients have not been developed yet.

Herein, we report a method for the human-like smelling of rose scent ingredients in real samples using olfactory receptor nanodisc (ND)-based bioelectronic nose devices. In this strategy, hOR1A2 was expressed from *Escherichia coli* (*E. coli*) and reconstituted using the ND structure. The hOR1A2-embedded nanodiscs (hOR1A2NDs) were successfully incorporated on gold (Au)-based floating electrodes (FEs) over a carbon nanotube (CNT) field effect transistor (FET). We could monitor the binding of target rose scent molecules onto the NDs via the subjacent CNT-based transistor. This method allowed us to identify and quantitatively monitor geraniol and citronellol, well-known rose odorants, down to 1 fM and 10 fM, respectively. This method can also be used to discriminate a specific rose odorant from other odorants just like a human olfactory system. Furthermore, we utilized our devices to quantitatively evaluate the effect of scent enhancer materials on the responses of ORs and found that when 1 nM benzyl salicylate was added, the ORs responded to rose odorants with ~10^3^ times lower concentrations. Importantly, the method enabled the quantitative evaluation of rose odorants in a real sample like rose oil. Since our method allows one to quantitatively evaluate general ingredients providing a rose scent even in real samples, it could be a powerful tool for versatile basic research and industrial applications such as the screening of new rose scent ingredients and the quantitative evaluation of base materials for fragrances.

## Methods

### Reagents and materials

A pMSP1E3D1 bacterial expression vector and lipids were purchased from Addgene (USA) and Avanti Polar Lipids (USA), respectively. Semiconducting 99% single-walled carbon nanotubes (CNTs) were purchased from NanoIntegris, Inc. (USA). Geraniol, R-( + )-citronellol, benzyl salicylate, 1-phenylethanol, linalool, α-damascone, geranyl chloride, geranyl formate, 1,7-octadiene, 3,7-dimethyl-1-octanol, amyl butyrate, trimethylamine, and other chemical reagents used in our experiments were purchased from Sigma Aldrich (USA). The natural rose oil (Damask rose) was provided by the team manager of LG Household & Health Care (Korea), Hoodeok Kim.

### Cloning of hOR1A2 into an expression vector

The cloning procedure of hOR1A2 was similar to that in a previous report^19^. The hOR1A2 genes were amplified by polymerase chain reaction (PCR) using human genomic DNA. The PCR products were cloned into a pcDNA3 mammalian expression vector and pET-DEST42 bacterial expression vector by enzyme methods and a gateway cloning system (Invitrogen, USA).

### Luciferase assay of hOR1A2 in human embryonic kidney-293 cells

Luciferase assays were performed in a similar way to a previous study^19^. Human embryonic kidney (HEK)−293 cells were grown in Dulbecco’s Modified Eagles Medium (DMEM) (HyClone, USA) supplemented with 10% Fetal Bovine Serum (FBS) (Gibco, USA), 1% streptomycin (Gibco, USA), and 1% penicillin at 37 °C under 5% CO_2_. The cells were transfected with a DNA mixture containing hOR1A2, G_αolf_, RTP1S, pCRE-Luc, and pSV40-RL using a Lipofectamine 3000 (Invitrogen, USA) according to a protocol provided by the manufacturer. The Dual-Glo Luciferase Assay System (Promega, USA) was used to measure the responses of hOR1A2 to various odorants. The HEK-293 cells expressing hOR1A2 were stimulated with serum-free DMEM, and then incubated in solutions containing various odorants. Luminescence intensities were measured using a Spark 10 M multimode microplate reader (TECAN, USA). A normalized luminescence intensity was calculated using a formula. In this measurement, 10 μM FSK and odorant-free DMEM solutions were used as a positive and negative control, respectively.

### Expression and purification of hOR1A2

The expression and purification of hOR1A2 were performed in a similar way to a previous report^19^. BL21 (DE3) *E. coli* cells were transformed by a pET-DEST42/hOR1A2 vector. Then, they were incubated in Luria-Bertani (LB) medium with 50 μg/mL ampicillin at 37 °C until an optical density (OD_600_) value reached 0.5. The induction of hOR1A2 protein was performed by the addition of 1 mM isopropyl thiogalactoside (IPTG), and then they were incubated for 4 h. After the incubation, the pellets of cells were centrifuged at 7000 g for 20 min at 4 °C. The cells were resuspended in phosphate buffered saline (PBS) containing 2 mM EDTA. The cells were sonicated for 5 min (5 s on/off). The cells were successively centrifuged at 12000 g for 20 min at 4 °C. Then, the pellets of the sample were solubilized with solubilization buffer (0.1 M Tris-HCl, 20 mM sodium dodecyl sulfate (SDS), 1 mM EDTA, and 100 mM dithiothreitol (DTT), pH 8.0) at 25 °C. The solubilized hOR1A2 was dialyzed against a 0.1 M sodium phosphate solution (pH 8.0) containing 10 mM SDS. Afterward, the hOR1A2 was filtered by a bottle top filter (pore size 0.45 μm, Thermo Scientific). After that, the hOR1A2 was loaded to a Ni affinity column equilibrated with a buffer solution (0.1 M sodium phosphate, 10 mM SDS, pH 8.0). The column was washed with the buffer solution (0.1 M sodium phosphate, 10 mM SDS) using a pH gradient (pH 8.0 to 7.0). The hOR1A2 was also eluted with the same buffer at pH 6.0. The purified hOR1A2 was dialyzed against HEPES buffer I (20 mM HEPES-NaOH, 100 mM NaCl, 25 mM cholate and 1 mM EDTA, pH 8.0).

### Expression and purification of MSP1E3D1

The expression of MSP1E3D1 was performed by following the method of hOR1A2 expression using the pMSP1E3D1 bacterial expression vector^19^. After the production of MSP1E3D1, the cells were harvested by a centrifugation at 7000 g for 20 min at 4 °C. Then, they were resuspended in lysis buffer (20 mM Tris-HCl, 20 mM imidazole and 0.5 M NaCl, pH 8.0), and they were sonicated for 5 min (5 s on/off). The cell lysates were centrifuged at 12000 g for 30 min at 4 °C. The soluble MSP1E3D1 was filtered by a bottle top filter (pore size 0.45 μm). Then, the MSP1E3D1 was applied to a HisTrap HP column (GE Healthcare, Sweden) using fast protein liquid chromatography (FPLC) (GE Healthcare). The column was successively washed with washing buffer (20 mM Tris-HCl, 50 mM imidazole and 0.5 M NaCl, pH 8.0). The target protein was collected with elution buffer (20 mM Tris-HCl, 400 mM imidazole and 0.5 M NaCl, pH 8.0). The purified MSP1E3D1 was dialyzed against the HEPES buffer I using the HiTrap HP desalting column (GE Healthcare) and stored at 4 °C until used.

### Total protein assays and western blot analysis

The concentrations of purified proteins were measured by a BCA assay kit (Pierce, IL, USA) using BSA protein as a standard, as reported previously^19^. All protein samples were analyzed by a sodium dodecyl sulfate polyacrylamide gel electrophoresis (SDS-PAGE) method and western blot analysis, as previously reported^19^. The western blot analysis was conducted using an anti-v5 epitope mouse antibody (Ab) (Santa Cruz Biotechnology, USA) and anti-His-probe mouse Ab (Santa Cruz Biotechnology, USA) as a primary Ab, respectively. A HRP-conjugated anti-mouse Ab (Milipore, USA) was used as a secondary Ab. For the detection of proteins, Luminata Forte Western HRP substrate (Millipore, USA) was used. The films were scanned using a G:BOX Chemi XL system (Syngene, UK), and the digitalized images were cropped and edited using PowerPoint and PhotoShop.

### Construction of hOR1A2NDs

The construction process of NDs was similar to that in a previous work^19^. Lipids (Palmitoyloleoylphosphatidylcholine (POPC), palmitoyloleoylphosphatidylglycerol (POPG)) were mixed in chloroform at the molar ratio of 1:1. Then, they were dried by nitrogen gas and put in a vacuum for 1 h to eliminate remaining chloroform. The lipids were solubilized with the HEPES buffer I, and they were added to the purified hOR1A2. The lipid/receptor mixture was incubated on ice for 10 min, and successively mixed with MSP1E3D1. The mixed solutions were incubated with a gentle stir for 2 h at 4 °C. The final mixture contained 1 μM hOR1A2, 8 mM lipids, 25 mM detergents, and 100 μM MSP1E3D1. Afterward, to adsorb detergents, Bio-Beads (Bio-Rad, USA) were applied to the mixed solution with agitation overnight. Lastly, the mixture was loaded to size exclusion chromatography (SEC) (Superdex 200 Increase 10/300 GL, GE Healthcare, USA) to remove unbound units. The column was equilibrated with HEPES buffer II (20 mM HEPES-NaOH, 100 mM NaCl and 1 mM EDTA, pH 8.0). The hOR1A2NDs were collected and stored at 4 °C.

### DLS analysis and SEM imaging

The size distribution of hOR1A2NDs was measured by a dynamic light scattering (DLS) spectrophotometer (DLS-7000, Japan). The forms of the NDs were observed by utilizing a field emission scanning electron microscope (FE-SEM) (SUPRA 55VP, Carl Zeiss, Germany).

### Fabrication process of FE-based CNT transistors

Floating electrode (FE)-based CNT transistors were fabricated following the method reported previously^20,21^. To disperse CNTs, CNTs were mixed with 1,2-dichlorobenzene (0.05 mg/mL) and sonicated for 5 h. For the assembly of CNTs, an octadecyltrichlorosilane (OTS) self-assembled monolayer with nonpolar terminal groups was formed on a SiO_2_ substrate (3000 Å) via photolithography. The patterned substrate was immersed into the CNT suspension for 20 s and washed thoroughly with 1,2-dichlorobenzene. Then, source, drain, and five floating electrodes (Pd/Au 10 nm/15 nm) were fabricated via photolithography and thermal evaporation. Each FE has a width of 200 μm and a length of 10 μm. The exposed CNT channel has a width of 3 μm and a length of 170 μm. Finally, a passivation layer (Photoresist, DNR) was formed using photolithography to prevent leakage currents during sensing measurement in aqueous environments.

### Measurement procedure for the liquid gate profiles of a CNT-FET

A FE-based CNT-FET was linked to a Keithley 4200 semiconductor analyzer. For the measurement of the liquid gating profiles of the CNT-FET, the 9 μL of distilled water was added on the exposed CNT channel. While maintaining the constant source-drain bias voltage (0.1 V), the source-drain currents were measured in a gate bias voltage range from −0.4 V to 0.4 V.

### Incorporation of hOR1A2NDs on the FEs of CNT-FETs

N-acetyl-L-cysteine powder was dissolved in distilled water to the final concentration of 0.5 M. A cysteine monolayer was formed on the surface of gold (Au)-based FEs by incubation in the N-acetyl-L-cysteine solution for 10 min at 37 °C. Then, the CNT-FETs were washed with distilled water. Subsequently, the FE-based CNT transistors were immersed in the solution of half-v5 Ab fragments. 2-Mercaptoethylamine hydrochloride (2-MEA) which is a reducing agent was used to divide the v5 Ab into two half-v5 Ab fragments^22^. After the incubation for 1 h at 37 °C, the CNT-FETs were washed with PBS buffer. The half-v5 Ab fragments were attached selectively on the surface of gold-based FEs via their thiol groups. Lastly, at room temperature, the attachment of hOR1A2NDs on the gold FEs was carried out by incubation in a solution containing hOR1A2NDs for 1 h. As a result, the hOR1A2NDs were incorporated only onto the gold-based FE surface of the CNT transistors using half-v5 Ab fragments and thiol groups as a linker.

### Preparation of odorants and natural rose oil solutions

Geraniol, R-( + )-citronellol, benzyl salicylate, 1-phenylethanol, linalool, α-damascone, geranyl chloride, geranyl formate, 1,7-octadiene, 3,7-dimethyl-1-octanol, amyl butyrate, trimethylamine, and other chemical reagents used in our experiments were dissolved in the HEPES buffer II. A 50 μL of the natural rose oil stock solution was mixed with 4.95 mL of the HEPES buffer II (1% v/v). The blended rose oil solution was filtered (syringe filter from Advantec, pore size 0.45 μm) and additionally diluted with the HEPES buffer II solution to prepare rose oil solutions diluted from 10^−11^ to 10^−5^.

### Electrical measurement

For the detection of the responses of a bioelectronic nose, the bioelectronic nose was coupled to the semiconductor analyzer. A 9 μL of the HEPES buffer II was put on the channel of the bioelectronic nose. In the electrical measurement, source-drain bias (0.1 V) was consistently maintained to the device. Current changes were measured upon the addition of various odorants and reagents.

### GC-MS analysis

The natural rose oil from Damask roses was analyzed by a gas chromatography mass spectrometry (GC-MS) (ISQ LT, USA) method. The compositions of the rose odorants in the rose oil were confirmed by comparing with authentic reference compounds.

## Results and Discussion

### Structure of a bioelectronic nose comprised of hOR1A2-based nanodiscs and a carbon nanotube-based transistor with floating electrodes

**Figure 1:**
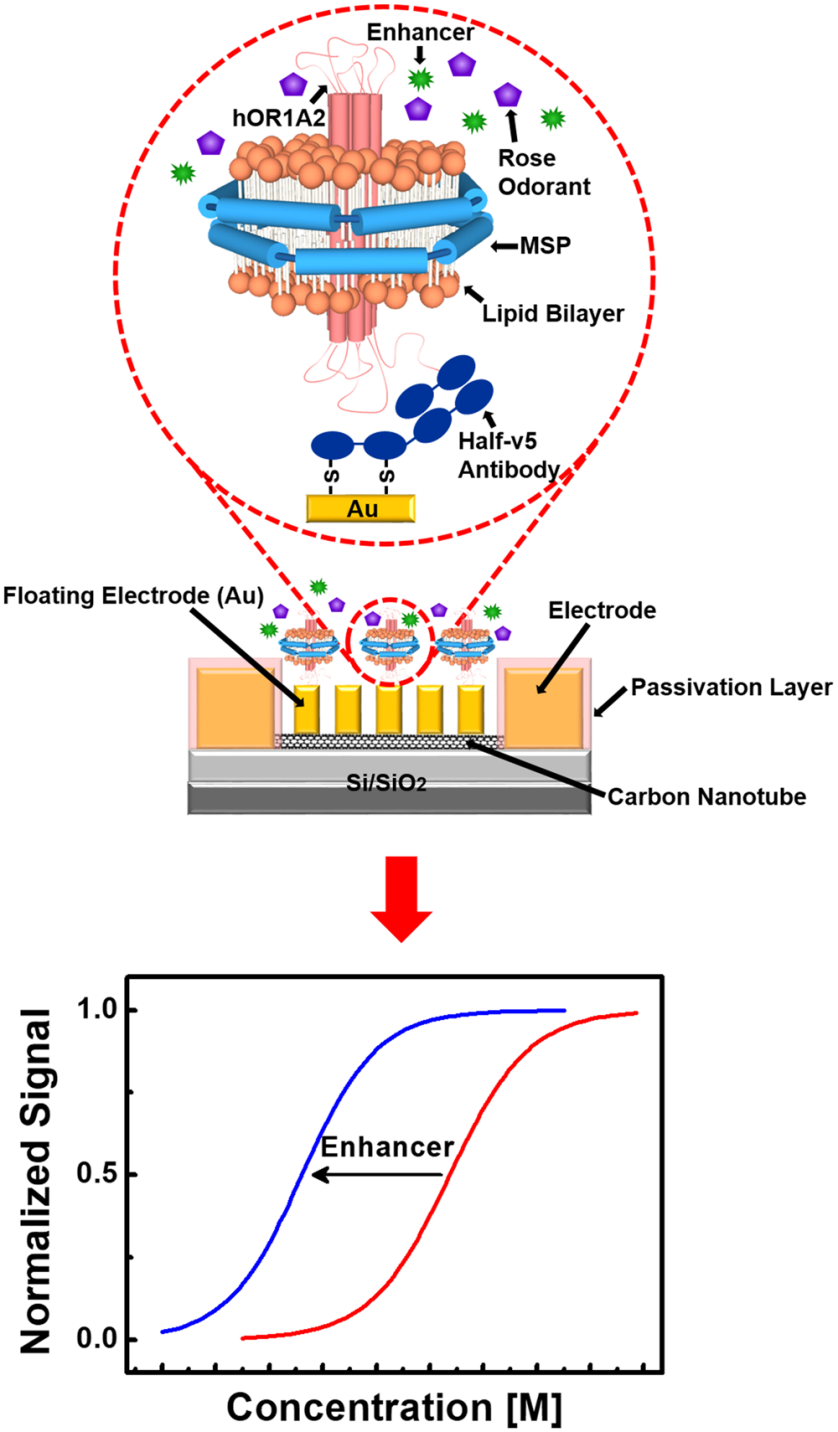
Schematic diagram depicting a bioelectronic nose based on the hybridization of hOR1A2-embedded nanodiscs (hOR1A2NDs) and a floating electrode-based CNT-FET, and the simplified sensor responses of a bioelectronic nose. The hOR1A2NDs were immobilized on the gold-based floating electrodes of the CNT-FET using half-v5 Ab fragments and thiol groups. The bioelectronic nose could specifically respond to general rose scent ingredients just like a human nose.

Figure 1 represents a schematic diagram depicting a bioelectronic nose which was fabricated via the hybridization of hOR1A2NDs and a CNT-based transistor with FEs. The detailed experimental procedures are described in the Methods section and Fig. S1 (Supplementary Information). In brief, hOR1A2 was overexpressed in *E. coli* and purified via affinity chromatography. Then, the purified hOR1A2 proteins were wrapped with lipid bilayers and membrane scaffold proteins (MSPs) for the construction of hOR1A2-based NDs. The prepared hOR1A2NDs were selectively immobilized on the flat gold FEs of the CNT-based transistor. Here, the gold FEs were first functionalized with thiol groups, and then the gold surfaces were functionalized with half-v5 antibody (Ab) fragments via disulfide bonding^19,22^. Subsequently, the hOR1A2NDs were incorporated on the gold-based FEs. Lastly, the CNT-FETs was washed with a HEPES buffer II solution several times to remove unbound hOR1A2NDs. When specific rose odorants bound to the hOR1A2 on the FE, the conductance of the CNT-FET channel was changed, which allowed us to monitor rose scent ingredients in real-time. In this fabrication strategy, receptor molecules stabilized in ND structures were directly and directionally immobilized on a flat gold-based FE surface using well-known reliable chemical processes, which should simplify the chemical procedures and may enhance the reliability of our devices compared with previous CNT channel-based sensor devices^15,23^. Furthermore, we can take advantage of the high sensitivity of FE-based transducers as reported previously^21^. Finally, since the hOR1A2 molecules bind selectively to general rose odorant molecules like a human nose, we can expect our sensors to smell a rose scent just like humans.

### Characterization of hOR1A2 expressed in HEK-293 cells

**Figure 2:**
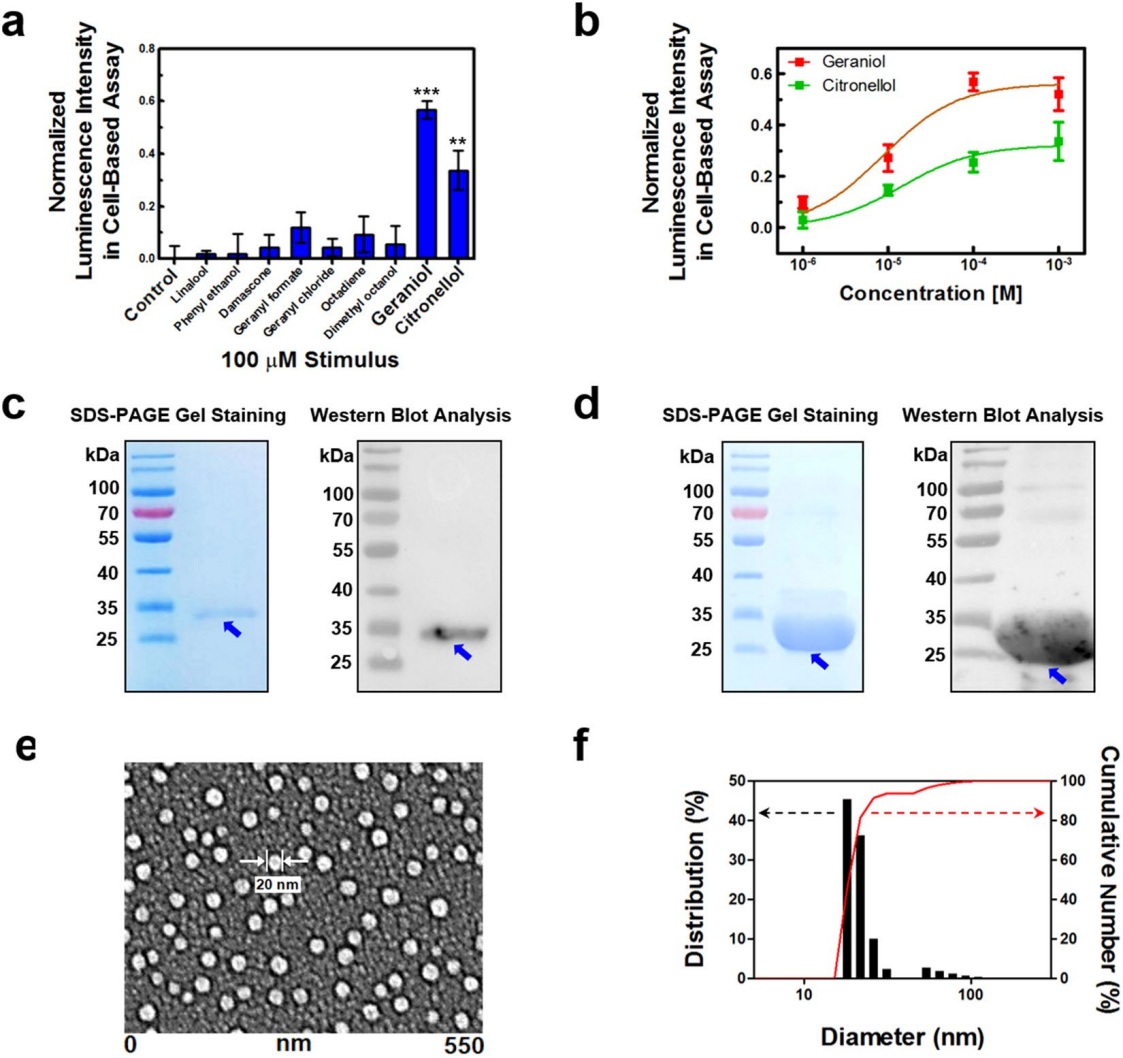
Characterization of hOR1A2 expressed in HEK-293 cells and NDs. (**a**) Specificity of hOR1A2 to geraniol and citronellol among various odorants. Only the stimulation of geraniol and citronellol caused responses in HEK-293 cells with hOR1A2 (**p < 0.01, ***p < 0.001) (n = 5). (**b**) Dose-dependent responses of hOR1A2 expressed in HEK-293 cells upon the addition of geraniol and citronellol. The HEK-293 cells expressing hOR1A2 exhibited luminescence intensities to geraniol and citronellol with different characteristics (n = 5). (**c**) SDS-PAGE gel staining image and western blot analysis of purified hOR1A2 expressed in *E. coli*. The band of 34 kDa indicates the molecular weight of hOR1A2. (**d**) SDS-PAGE gel staining image and western blot analysis of purified MSP1E3D1 expressed in *E. coli*. The band of 26 kDa corresponding to the molecular weight of MSP1E3D1 was observed. (**e**) FE-SEM image of hOR1A2NDs immobilized on a gold substrate. The NDs were immobilized uniformly on the gold surface, and their diameters ranged from 15 nm to 20 nm. (**f**) Size distribution analysis of hOR1A2NDs. The gels and blots presented in (**c**–**d**) were cropped from different images to improve clarity. The black lines surrounding blots and gels indicate the cropping lines. Full-length gels and blots are presented in Supplementary Figure S6.

We performed cell-based assays to evaluate the ligand/receptor binding activity of hOR1A2 in cells and to compare it with the binding activity of hOR1A2 in our bioelectronic nose devices^24^. Figure 2a shows the responses of hOR1A2 in HEK-293 cells to various kinds of odorants with 100 μM concentrations. Detailed procedures for the cell-based assay are described in the Methods section. In brief, hOR1A2 and luciferase gene were first transfected in the HEK-293 cells. When the binding of the specific odorants to hOR1A2 in the transfected cells occurred, the conformation of hOR1A2 was changed and signal transduction was initiated. A cyclic adenosine monophosphate (cAMP) pathway in the cells was sequentially activated. The activated cAMP pathway stimulated cAMP response element-binding protein (CREB), which resulted in the expression of luciferase gene^25^. A luminescence intensity in the cells was measured using a Dual-Glo luciferase assay system after the introduction of different odorants. A normalized luminescence intensity was calculated by normalizing the responses of hOR1A2 to each odorant with respect to a positive control (forskolin, FSK) in HEK-293 cells (see the details in the Methods section). Linalool, phenyl ethanol, and damascone have pleasant floral odors. They are often used to make an artificial rose scent with geraniol and citronellol. Geranyl formate and geranyl chloride have different functional groups from geraniol. Octadiene and dimethyl octanol have different numbers of carbon atoms and carbon-carbon double bonds compared with geraniol, respectively. The HEK-293 cells expressing hOR1A2 stimulated by geraniol and citronellol resulted in much higher luminescence intensities compared to those stimulated by other odorants. These results show that hOR1A2 discriminates the specific rose odorants from other odorants with a high selectivity, indicating that hOR1A2 was well expressed and retained its functionality in the cells.

Figure 2b displays the dose-dependent responses of hOR1A2-expressing HEK-293 cells to geraniol and citronellol. The responses of hOR1A2 in the HEK-293 cells to different concentrations of geraniol and citronellol were measured by the luciferase assay system in a similar way to that of Fig. 2a (see the details in the Methods section). The response data were fitted using a Hill equation to evaluate dissociation constants (*K*_*d*_) and Hill coefficients. The HEK-293 cells expressing hOR1A2 exhibited luminescence intensities to geraniol and citronellol from the concentrations of 1 μM and 10 μM, respectively. The dissociation constants (*K*_*d*_) of hOR1A2 to geraniol and citronellol were calculated as 3.24 × 10^−6^ M and 1.45 × 10^−5^ M, respectively. These constant values are quite similar to those in other studies using mammalian cell-based systems^26,27^. The results imply that functional hOR1A2 was successfully produced in the HEK-293 cells, while maintaining its functionality. Also, we can see that the receptors in the cells exhibited larger responses to geraniol than citronellol, presumably due to the higher affinity of hOR1A2 to geraniol than citronellol. The Hill coefficients were estimated as *0.55* and *0.52* for *geraniol* and *citronellol*, respectively. This indicates that the binding of geraniol and citronellol to hOR1A2 could be considered as negatively cooperative bindings^28^. Previous works show that for most GPCRs, the binding of one ligand to one binding site causes the structural change of neighboring binding sites, which may lead to a lower affinity for other ligands like our results^29–31^.

### Reconstitution of hOR1A2 into nanodiscs

For the development of bioelectronic nose devices smelling a rose scent, we expressed hOR1A2 in *E. coli* and performed the extraction and functional reconstruction of hOR1A2 molecules in solution^19,32,33^. Figure 2c shows the sodium dodecyl sulfate polyacrylamide gel electrophoresis (SDS-PAGE) gel staining image (left) and western blot analysis (right) of purified hOR1A2 expressed in *E. coli*. For the formation of high-quality receptor proteins, hOR1A2 was overexpressed in *E. coli*, solubilized and purified with affinity chromatography. The purification and expression of hOR1A2 in *E. coli* were confirmed by a SDS-PAGE method and western blot analysis (see the details in the Methods section). The bands of 34 kDa, which are in accord with the molecular weight of hOR1A2, were clearly observed. These results indicate that hOR1A2 was well produced in *E. coli* and highly purified. It should be pointed out that it has been very difficult to express and purify G protein-coupled receptors (GPCRs) in heterologous cells, especially in *E. coli* due to their complicated structures and hydrophobicity. Such a difficulty has been a stumbling block holding back the practical applications of OR-based biosensor devices^34,35^. Considering that the successful expression of hOR1A2 in *E. coli* has not been reported before, our results can be a breakthrough and could provide more opportunities for biosensors and other applications requiring a large amount of high-quality OR proteins responding to a rose scent.

To achieve a stable OR functionality on our bioelectronic nose devices, hOR1A2 was embedded in ND structures. First, MSP1E3D1, which is MSP derived from apolipoprotein A-I in humans, was produced and purified to wrap lipid/receptor complexes^36^. Figure 2d shows the SDS-PAGE gel staining image (left) and western blot analysis (right) of purified MSP1E3D1. The expression and purification of MSP1E3D1 were confirmed in a similar way to that of Fig. 2c (see the details in the Methods section). The thick bands around 26 kDa were clearly observed. The bands correspond to the molecular weight of MSP1E3D1. These indicate that MSP1E3D1 was overexpressed, successfully produced as a soluble form, and purified with high purity. Because it has been reported that MSP1E3D1 can be overexpressed in *E. coli* and its overexpression allows it to effectively wrap lipid/receptor complexes^37^, our results imply that MSP1E3D1 was successfully overexpressed to construct stable ND structures for the development of bioelectronic devices.

To construct hOR1A2-embedded NDs, the purified MSP1E3D1 was added to the mixture of hOR1A2 and lipids. Then, the detergent molecules of the mixture were removed with Bio-beads. The purified hOR1A2NDs were finally obtained by size exclusion chromatography (SEC). Figure 2e shows the field emission scanning electron microscopy (FE-SEM) image of hOR1A2NDs incorporated on the surface of a gold. To maintain the structure of hOR1A2NDs, the hOR1A2NDs immobilized on the gold surface were lyophilized using a freeze dryer. Then, the surface was covered with platinum (5 nm) by a sputtering system. The hOR1A2NDs ranged in diameter from 15 nm to 20 nm. This clearly shows that we successfully constructed hOR1A2NDs with optimized sizes and could immobilize them uniformly on gold surfaces.

To confirm the size distribution of the constructed hOR1A2NDs, we performed a dynamic light scattering (DLS) analysis (Fig. 2f). The hOR1A2NDs had uniform diameters from 15 nm to 20 nm with a quite narrow size distribution, which is similar to that of Fig. 2e. The size distribution of hOR1A2NDs is also close to that of NDs containing other GPCRs in previous studies^38,39^. This implies that hOR1A2NDs can be successfully constructed as monomeric receptor forms and can be utilized as an ideal sensor unit.

### Electrical characterization of CNT-FETs

**Figure 3:**
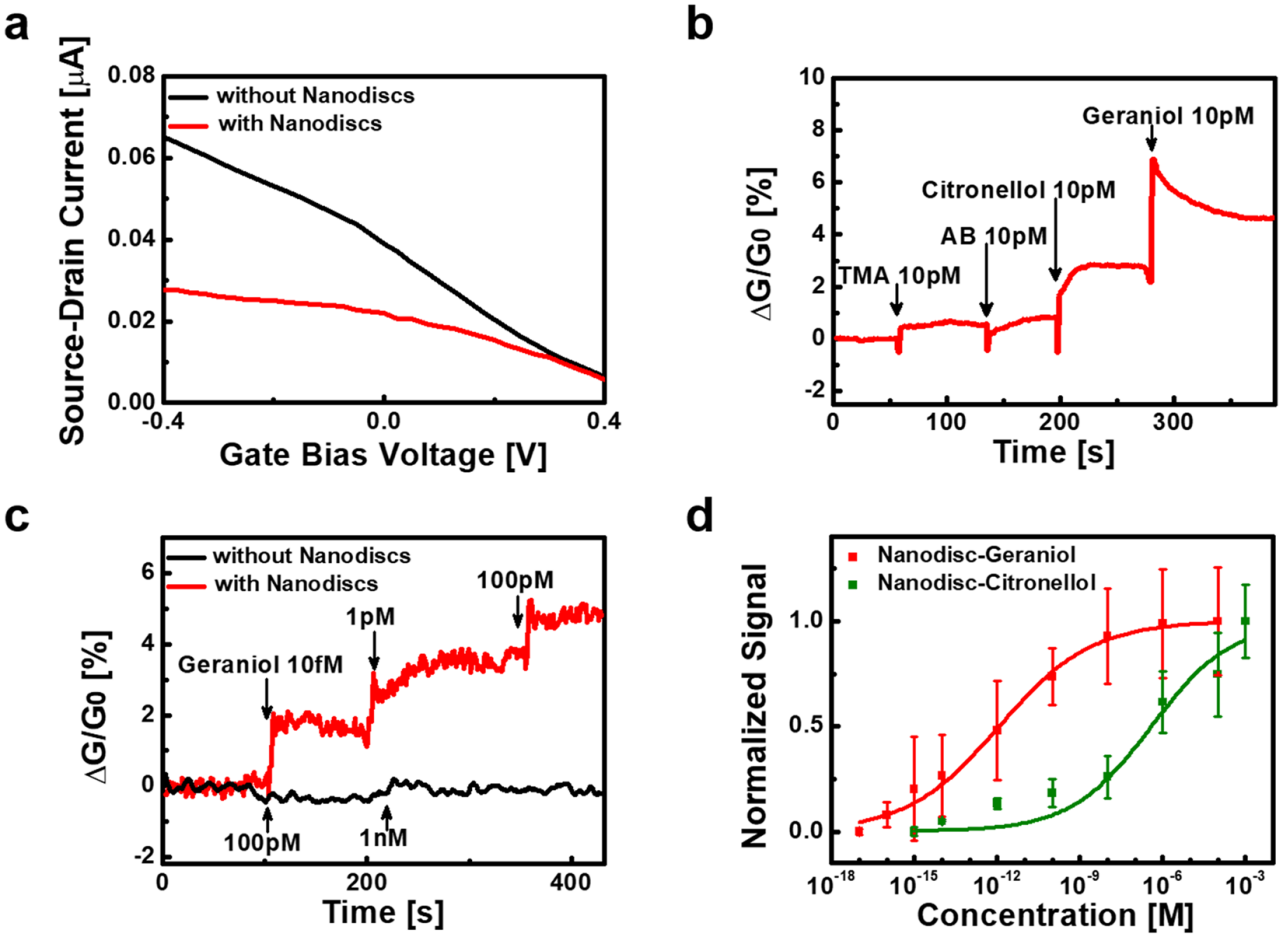
Electrical measurement data of ND-based bioelectronic noses. (**a**) Liquid gate profiles of a CNT-FET with floating electrodes before and after the immobilization of hOR1A2NDs. The CNT-FET with floating electrodes exhibited a typical p-type semiconducting property, and its characteristic was maintained after the immobilization of hOR1A2NDs. (**b**) Real-time responses of a bioelectronic nose to different kinds of odorants. The addition of geraniol and citronellol of 10 pM concentrations caused significant increases in the CNT-FET channel conductance, while the addition of TMA and AB of 10 pM concentrations resulted in negligible changes in the CNT-FET channel conductance. (**c**) Real-time responses of a bioelectronic nose device with or without hOR1A2NDs to various concentrations of geraniol. The introduction of geraniol occurred sharp increases in the channel conductance of the bioelectronic nose, while there was no meaningful conductance change in the bare CNT-FET without NDs. (**d**) Dose-dependent responses of bioelectronic noses to geraniol and citronellol. Each point and error bar represent the average value and standard deviation of multiple sensing measurements, respectively. Bioelectronic noses began to show responses to geraniol with the concentration of 1 fM, and the responses were almost saturated at around 1 μM. Bioelectronic noses also exhibited the responses from 10 fM concentration of citronellol.

Figure 3a shows the liquid gate profiles of a FE-based CNT transistor before and after the immobilization of hOR1A2NDs. Source-drain currents were measured at a gate bias voltage ranging from −0.4 V to 0.4 V with the application of 0.1 V source-drain bias voltage. The source-drain currents decreased significantly as the applied gate voltage increased, indicating the typical p-type semiconducting behavior of the CNT-FET device. Note that the conductance of the CNT-FET channel decreased after the immobilization of hOR1A2NDs, which could be attributed to the negatively charged C-terminuses of the NDs immobilized on the FEs^40^. The negatively-charged NDs reduced the CNT channel conductance, which was attributed to Schottky barrier modulation at the CNT-FE contacts. Also, it should be mentioned that the gating effect of the CNT-FET device was maintained even after the immobilization of hOR1A2NDs, indicating that it can be suitable for sensor applications^41^.

### Electrical responses of bioelectronic noses to geraniol and citronellol

Figure 3b shows the real-time responses of a bioelectronic nose to various odorants. Trimethylamine (TMA) and amyl butyrate (AB) are odorants generated from spoiled seafood and reminiscent of an apricot, respectively. When the source-drain bias of 0.1 V was applied, source-drain currents were observed during the addition of different odorant solutions. In this sensor device, a relative CNT-FET channel conductance change ${\rm{\Delta }}G/{G}_{0}$ was used as a sensor signal, where ${\rm{\Delta }}G$ and ${G}_{0}$ are the *conductance change* and *original conductance* of the CNT-FET, respectively. The introduction of geraniol and citronellol solutions of 10 pM concentrations caused significant increases in the conductance of the CNT channel. However, the introduction of TMA and AB with the same concentrations induced negligible changes. This result indicates that our bioelectronic nose could highly selectively discriminate rose scent odorants from other odorants.

Figure 3c displays the typical real-time responses of a bioelectronic nose with or without hOR1A2NDs to varying concentrations of geraniol in aqueous environments. Source-drain currents were measured during the addition of geraniol solutions with different concentrations. The addition of geraniol solutions caused immediate increases in the conductance of the CNT-FET with hOR1A2NDs in a dose-dependent manner, while the bare CNT-FET without hOR1A2NDs did not exhibited conductance changes even after the addition of geraniol. We could obtain a similar selective response when citronellol was applied to CNT-FET devices with or without hOR1A2NDs (Fig. S2 in the Supplementary Information). The result clearly shows that the sensor responses came from the specific binding between the rose odorants and hOR1A2. Such a specific response can be attributed to the change of electrical charges in the hOR1A2 caused by the selective binding of rose odorant molecules. In brief, the specific binding of rose odorant molecules to hOR1A2 caused the conformational change of the receptor, resulting in the change of electrical charges in it^14^. Subsequently, the changed charge state of the receptor molecule would result in the increase in the conductance of the CNT channel via Schottky barrier modulation at CNT-FE contacts^20,21^. This result shows that our method can allow us to highly sensitively detect specific rose odorants in real-time.

Figure 3d displays the normalized sensor signals of our bioelectronic noses to geraniol and citronellol with different concentrations. The normalized signals were calculated by normalizing sensor signals regarding their maximal sensor signal values at high concentration conditions^21,42,43^. The sensing measurement at a single concentration was carried out repeatedly using multiple bioelectronic noses to obtain average values and standard errors. The result shows the response curves similar to those of other bioelectronic nose devices reported previously^18,41^. In the case of geraniol, our bioelectronic noses began to show responses from the concentration of 1 fM (signal-to-noise ratio of ~4.4, Fig. S3a in the Supplementary Information), and the responses were almost saturated around 1 μM. For citronellol, bioelectronic noses exhibited the responses from the concentration of 10 fM (signal-to-noise ratio of ~5.5, Fig. S3b in the Supplementary Information). These results indicate that our bioelectronic noses could detect the rose scent odorants with a high sensitivity and discriminate one rose scent odorant from the other odorant. It is worth discussing the effects of possible impurities in the chemicals used in our experiments because, at such a low target concentration, the effect of the impurities on sensor signals may be comparable to that by target molecules. First of all, the impurity content in the target chemicals containing target molecules was less than 5% in our experiments. Furthermore, when the target chemicals were diluted to lower concentrations, their impurities should also be diluted in the same proportion. Therefore, even though bioelectronic noses were stimulated by target chemicals with a very low concentration, the impurities should not affect sensing signals much. On the other hand, the impurity concentrations in a HEPES buffer solution, which was used as a solvent to prepare target solutions, should have remained identical even in target solutions with a very low target concentration. However, we found that our bioelectronic noses did not respond to the HEPES buffer solution without any target molecules, indicating that the effect by impurities is not significant even in the target solution with a very low target concentration.

The dose-dependent responses of our bioelectronic noses can be analyzed further using a model based on a Hill-Langmuir equation as reported previously^19,21,42,43^. If we suppose that binding characteristics between target odorant molecules (geraniol and citronellol) and receptors (hOR1A2) comply with the model, the surface density ${C}_{s}$ of the odorant molecules bound to hOR1A2 in the NDs can be simply written like

1 $${C}_{s}=\frac{{C}_{s\_max}\cdot {C}^{n}}{{(1/K)}^{n}+{C}^{n}}$$

*C* is the *concentration of the applied odorant solution*, and *K* is the *equilibrium constant for the binding of the odorants to hOR1A2*. ${C}_{s\_max}$ denotes the *density of hOR1A2 on the FEs*, and *n* represents a *Hill coefficient*. Assuming a conductance change ${\rm{\Delta }}G$ is nearly proportional linearly to the number of adsorbed odorant molecules, the sensor signal $|{\rm{\Delta }}G/{G}_{0}|$ could be simplified as $|{\rm{\Delta }}G/{G}_{0}| \sim k{C}_{s}$. Here, *k* represents a constant signifying the response characteristics of a bioelectronic nose. The sensor signal $|{\rm{\Delta }}G/{G}_{0}|$ converges to the maximum value of $\,k{C}_{s\_max}$ as *C* becomes very large. Therefore, a normalized signal *N* could be written like

2 $$N=\frac{{C}^{n}}{{(1/K)}^{n}+{C}^{n}}$$

The experimental data were fitted by Eq. (2), and the equilibrium constants of geraniol and citronellol to hOR1A2 were estimated as 8.37 × 10^11^ M^−1^ and 2.60 × 10^6^ M^−1^, respectively. Note that the equilibrium constant of geraniol was found ~10^5^ times larger than that of citronellol. The results imply that geraniol could be a more potent rose scent than citronellol, which is consistent with the result using cells in Fig. 2b. However, the results show that our bioelectronic noses responded to much lower concentrations of geraniol and citronellol than the case of the cell-based assays in Fig. 2b. Presumably, it is because our device could directly measure the conformation of receptor proteins without any intermediate biological steps, while, the cell assays relied on complicated signal transduction steps based on multiple biological processes caused by the binding of odorant molecules to receptors^44–47^. The intermediate steps in the cell assays require several different materials other than rose odorants to generate the sensing signals, which could have resulted in much lower sensitivity than the bioelectronic noses^34^. Similar trends were also reported in case of other bioelectronic devices^21,42^. The Hill coefficients *n* were estimated as 0.26 and 0.30 for geraniol and citronellol, respectively. This also indicates the negatively cooperative binding of geraniol and citronellol to hOR1A2 on bioelectronic noses^48^. Note that the Hill coefficients in bioelectronic noses were smaller than those in the cell-based assays as shown in Fig. 2b. Presumably, it is because of the possible aggregation and steric hindrance of receptor proteins on our bioelectronic nose devices^48^. Since our method is based on these bioelectronic noses, it can be a sensitive platform for versatile utilization such as the development of new fragrances.

### Measuring the effect of a scent enhancer on the assessment of rose scent ingredients

**Figure 4:**
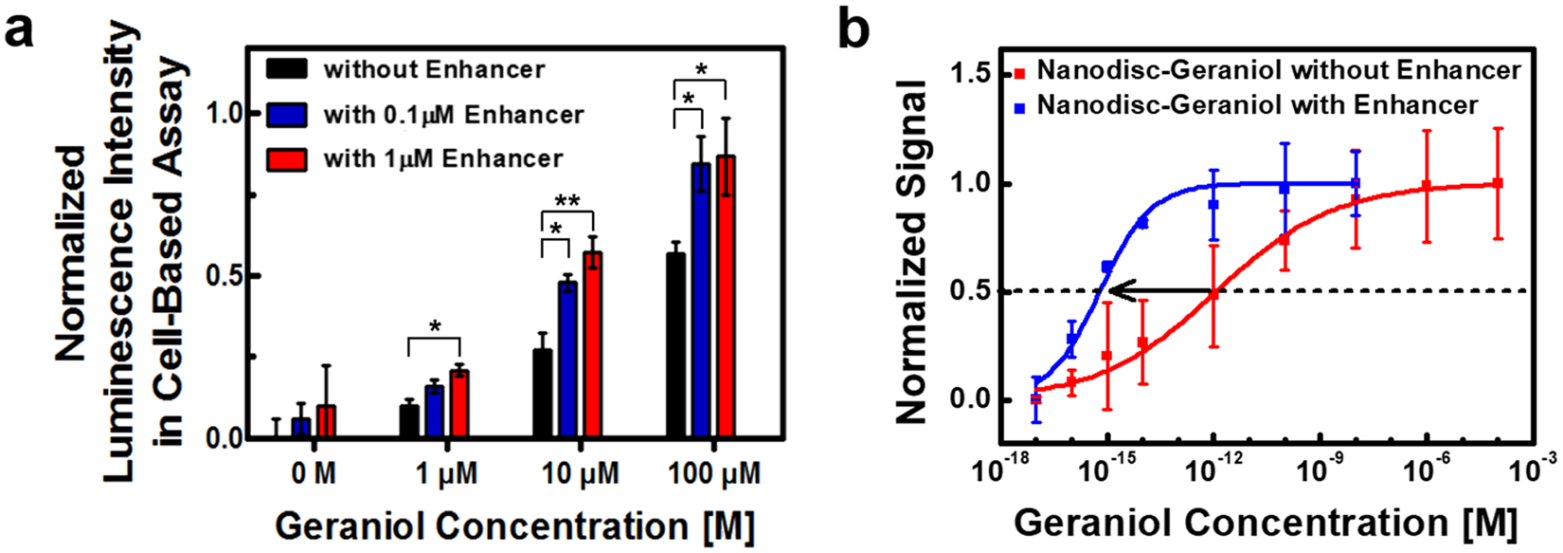
Effect of benzyl salicylate as an enhancer on the assessment of geraniol by utilizing hOR1A2-expressing HEK-293 cells and bioelectronic noses. (**a**) Normalized luminescence intensities of hOR1A2 to geraniol and benzyl salicylate in HEK-293 cells. The HEK-293 cells expressing hOR1A2 were activated by geraniol with or without varying concentrations of benzyl salicylate (0, 0.1, 1 μM). The responses of hOR1A2 to geraniol were enhanced by benzyl salicylate (*p < 0.05, **p < 0.01) (n = 5). (**b**) Normalized signals of bioelectronic noses at various concentrations of geraniol in the presence and absence of 1 nM benzyl salicylate. Each point and error bar represent an average value and standard deviation for multiple experiments, respectively. The equilibrium constant between hOR1A2 and geraniol in the absence of benzyl salicylate was estimated as 8.37 × 10^11^ M^−1^. In the presence of benzyl salicylate, the estimated equilibrium constant between hOR1A2 and geraniol was found to be 1.64 × 10^15^ M^−1^.

Previous works show that enhancer materials such as benzyl salicylate, which occurs naturally in various plants, could enforce the richness and depth of floral compositions^49,50^. However, the effect of such enhancers has not been evaluated quantitatively before. Here, we first performed cell-based assays to investigate whether the enhancer affects the responses of hOR1A2 in cells. Figure 4a is the cell assay results showing the responses of hOR1A2 to geraniol with or without an enhancer, benzyl salicylate. In brief, hOR1A2-expressing HEK-293 cells were activated by varied concentrations of geraniol in benzyl salicylate. When 0.1 μM benzyl salicylate was applied, the responses of hOR1A2 to *1*, *10* and *100* *μM* geraniol were found to be enhanced by *1.63-fold*, *1.76-fold* and *1.48-fold* compared with those without the enhancer, respectively. Likewise, benzyl salicylate with a 1 μM concentration led to *2.14-fold*, *2.09-fold* and *1.52-fold* enhancement in the responses of hOR1A2 to *1*, *10* and *100* *μM* geraniol, respectively. In addition, benzyl salicylate alone did not stimulate hOR1A2. These results imply that benzyl salicylate could enhance the responses of ORs to their floral scent molecules and thus enables low detection thresholds to them. Previous works show that the responses of some ORs to their odorants could be enhanced by enhancer materials such as benzyl salicylate via the allosteric modulation mechanism^51,52^. To our knowledge, this result is the first demonstration that benzyl salicylate could highly boost the responses of the cells expressing ORs to the specific odorant.

We also investigated the effect of the enhancer on the assessment of geraniol using our bioelectronic noses. Figure 4b shows the normalized signals of our bioelectronic noses to geraniol with or without 1 nM benzyl salicylate. First, we prepared the mixture of geraniol and benzyl salicylate, holding the concentration of benzyl salicylate at 1 nM and varying the concentrations of geraniol from 10 aM to 10 nM. Each data point was obtained by multiple measurements using four or more bioelectronic nose devices. Note that the normalized signal curve in the presence of benzyl salicylate was shifted to lower concentration regions, indicating that the bioelectronic noses began to exhibit responses at much lower concentrations of geraniol than the cases without the enhancer. We also confirmed that benzyl salicylate alone did not respond to a bare FE-based CNT transducer (Fig. S4 in the Supplementary Information). Following the Hill-Langmuir equation as described above, we could estimate the equilibrium constants between hOR1A2 and geraniol with or without benzyl salicylate. In the presence of 1 nM benzyl salicylate, the equilibrium constant between hOR1A2 and geraniol was estimated to be *1.64* × *10*^*15*^ *M*^*−1*^, while that without the enhancer was *8.37* × *10*^*11*^ *M*^*−1*^ in the section of Fig. 3d. These results clearly show that benzyl salicylate contributes significantly to the enhancement of the hOR1A2 responses to geraniol and thus decreases the thresholds of binding between hOR1A2 and geraniol. This is the first report showing that benzyl salicylate as the enhancer could affect the binding affinity of the receptor on bioelectronic devices. It also should be noted that since our method directly measured the responses of receptors without relying on complicated signal pathways like cell assays, it can be a powerful method to quantitatively evaluate the effect of enhancer materials on the binding of rose odorant molecules to receptors. Such a capability of our method could open up versatile applications in various areas such as drug, food, and cosmetic industries.

### Quantitative measurement of rose scent ingredients in natural rose oil

**Figure 5:**
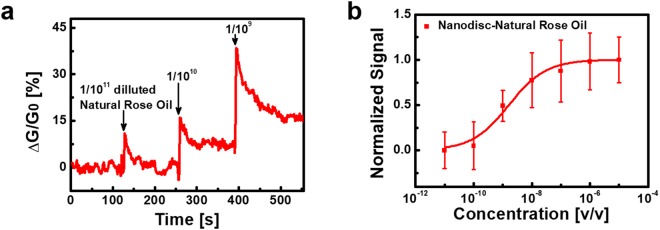
Quantitative measurement of rose scent ingredients in natural rose oil utilizing bioelectronic noses. (**a**) Real-time responses of a bioelectronic nose to different concentrations of natural rose oil. The introduction of the rose oil solution diluted by 10^−11^ occurred negligible responses in the conductance of the bioelectronic nose. The rose oil solutions diluted by 10^−10^ and 10^−9^ caused significant increases in the CNT-FET channel conductance. (**b**) Normalized signal of bioelectronic noses to natural rose oil solutions diluted from 10^−11^ to 10^−5^. The x-axis (v/v) represents the volume/volume percent of the natural rose oil in the HEPES buffer solution. We repeated the sensing measurement using four or more bioelectronic noses to obtain average values and standard deviation.

Rose oil products extracted from roses have been utilized as base materials for versatile applications such as perfumes^53–55^. In this case, the quantitative evaluation of rose scent ingredients in rose oil can be important because the overdose of the ingredients may result in bad smells^56^. To demonstrate the validity of our method for practical applications, we performed experiments to quantitatively evaluate rose scent ingredients in natural rose oil by utilizing our method. Figure 5a shows the responses of a bioelectronic nose to different concentrations of natural rose oil in real-time. The rose oil solutions were prepared by means of dilution of a natural rose oil stock solution with HEPES buffer II (see the details in the Methods section). The diluted rose oil solutions were consecutively introduced to the bioelectronic nose, and its responses were monitored simultaneously. The rose oil diluted by 10^−11^ caused negligible responses in the conductance of the bioelectronic nose. However, those diluted by 10^−10^ and 10^−9^ led to significant increases in the CNT-FET channel conductance. This result clearly shows that the bioelectronic nose could detect the specific rose compounds in real samples such as natural rose oil.

Figure 5b shows the normalized signal of bioelectronic noses to the natural rose oil diluted for different concentrations. The rose oil stock solution was serially diluted with HEPES buffer II to prepare rose oil solutions diluted from 10^−11^ to 10^−5^. We obtained the normalized signal of bioelectronic noses to the diluted rose oil solutions by fitting the response data in the same way as shown in Fig. 3d. We repeated measurement for four or more bioelectronic noses to obtain quantitative results. Since the bioelectronic noses detect general species which bind to hOR1A2, our strategy can efficiently evaluate general ingredients giving a rose scent. We confirmed that geraniol could be more dominant to hOR1A2 than citronellol by the results of Figs 2b and 3d. That is, hOR1A2 of the bioelectronic nose responds to geraniol mainly. This indicates that geraniol could play a dominant role in the responses of bioelectronic noses to the natural rose oil^57,58^. Then, we could estimate the concentration of geraniol in the undiluted rose oil solution by comparing *K*_*rose oil*_ with *K*_*geraniol*_, where *K*_*rose oil*_ and *K*_*geraniol*_ are the dissociation constants of hOR1A2 to the diluted rose oil and geraniol, respectively. The *K*_*rose oil*_ was found to be 1.62 × 10^−9^ from the normalized signal of bioelectronic noses to the diluted rose oil solutions. Likewise, the *K*_*geraniol*_ was calculated to be 1.19 × 10^−12^ M from the data in section of Fig. 3d. On the basis of comparison of *K*_*rose oil*_ to *K*_*geraniol*_, we could estimate the concentration of geraniol in the undiluted rose oil as about 7.35 × 10^−4^ M. This value is quite close to geraniol concentration estimated by a gas chromatography mass spectrometry (GC-MS) method (Fig. S5 in the Supplementary Information). The concentration of geraniol in the undiluted rose oil was estimated as 9.47 × 10^−4^ M by the GC-MS analysis. This means that the binding affinity of hOR1A2 with geraniol could be slightly underestimated in complicated environments containing various chemicals which could lead to lower effective concentration of geraniol to the receptor^59^. This result clearly shows that the bioelectronic noses could recognize geraniol in complex environments such as real rose oil. Also, this result indicates that the bioelectronic nose could be utilized for practical applications, which could open up various applications such as a stable and reliable sensor platform.

In summary, we have developed an olfactory receptor ND-based bioelectronic nose which can smell rose scent ingredients in real samples like a human nose. In this method, we directly incorporated hOR1A2NDs onto the gold FEs of a CNT-based transistor. The binding events between hOR1A2NDs and specific rose scent components were monitored electrically by the underlying CNT-FET. Using this method, we could quantitatively recognize geraniol and citronellol down to 1 fM and 10 fM, respectively. Additionally, the method allowed us to distinguish a specific rose odorant from other odorants with a high selectivity. Most noticeable, our sensors were utilized to investigate the effect of the scent enhancer on the responses of ORs, and we found that the ORs in the presence of 1 nM benzyl salicylate responded to a rose scent with ~10^3^ times lower concentrations. Furthermore, the method facilitated the quantitative detection of rose odorants in real rose oil just like a human nose. These results clearly show that the strategy could be a simple but powerful impetus for basic research and various applications in perfume and cosmetic industries.

## Electronic supplementary material

Supplementary Information
