# Supplementary material for: Human-like smelling of a rose scent using an olfactory receptor nanodisc-based bioelectronic nose
Source: Sci Rep. 2018 Sep 17;8:13945. doi: 10.1038/s41598-018-32155-1 (PMC6141559; doi:10.1038/s41598-018-32155-1)
Supplement: Supplementary file 1 — Supplementary Information [file 41598_2018_32155_MOESM1_ESM.docx]

[Supplementary Information]

**Human-like smelling of a rose scent using an olfactory receptor nanodisc-based bioelectronic nose**

Minju Lee^1^, Heehong Yang^2,3^, Daesan Kim^4^, Myungjae Yang^1^, Tai Hyun Park^2*^ & Seunghun Hong^1*^

^1^Department of Physics and Astronomy, and Institute of Applied Physics, Seoul National University, Seoul 08826, Korea

^2^School of Chemical and Biological Engineering, Seoul National University, Seoul 08826, Korea

^3^Protein Engineering Laboratory, Recombinants Unit, MOGAM Institute for Biomedical Research, Yongin 16924, Korea

^4^Department of Biophysics and Chemical Biology, Seoul National University, Seoul 08826, Korea

*Email: seunghun@snu.ac.kr (S. Hong), thpark@snu.ac.kr (T.H. Park)

^
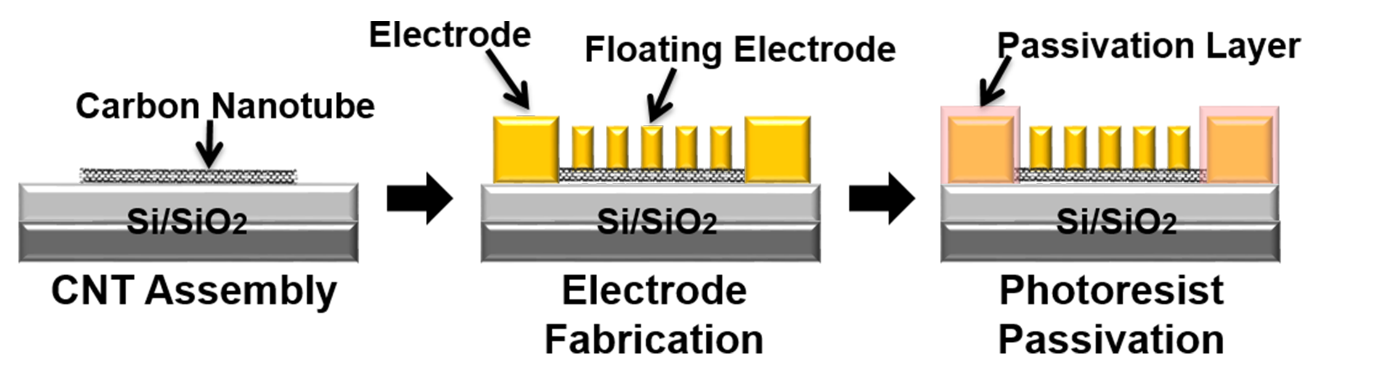
^

**Figure S1.** Schematic diagram depicting the fabrication processes of a CNT-FET with floating electrodes. First, CNTs were selectively assembled on a SiO_2_ substrate. Following the CNT assembly method, source, drain, and floating electrodes were fabricated using photolithography and thermal evaporation. Finally, a passivation layer was formed on the source and drain electrodes.


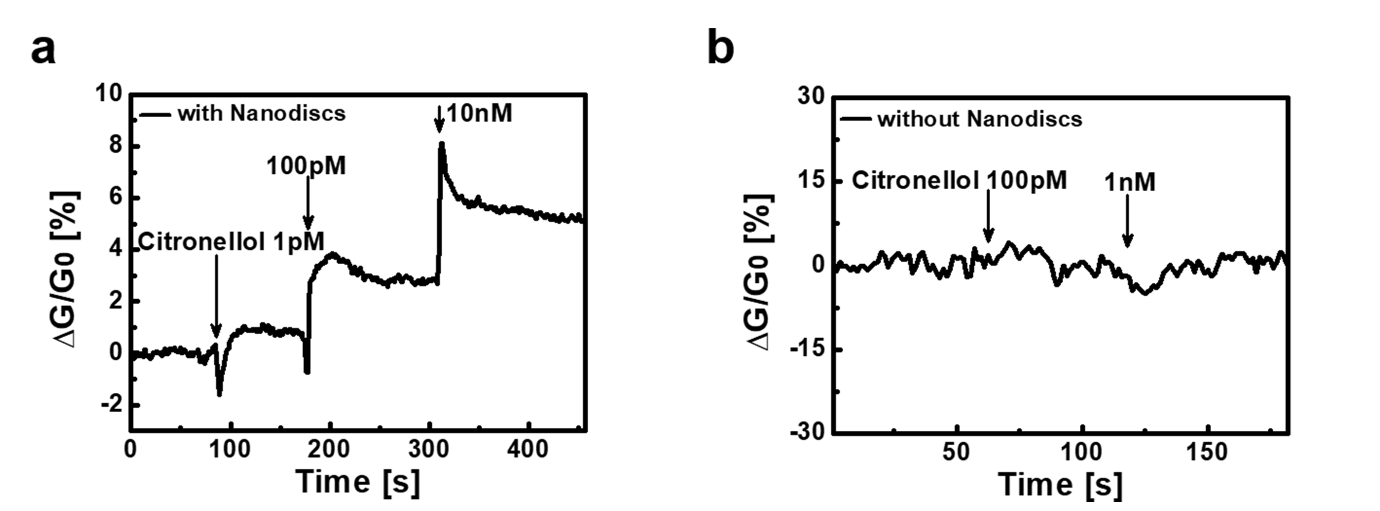


**Figure S2.** (a) Real-time responses of a ND-based bioelectronic nose to different concentrations of citronellol. The addition of citronellol resulted in the sharp increases in the CNT-FET channel conductance. (b) Real-time responses of a bare CNT-FET without hOR1A2NDs to various concentrations of citronellol. The bare CNT-FET without hOR1A2NDs did not respond to citronellol.


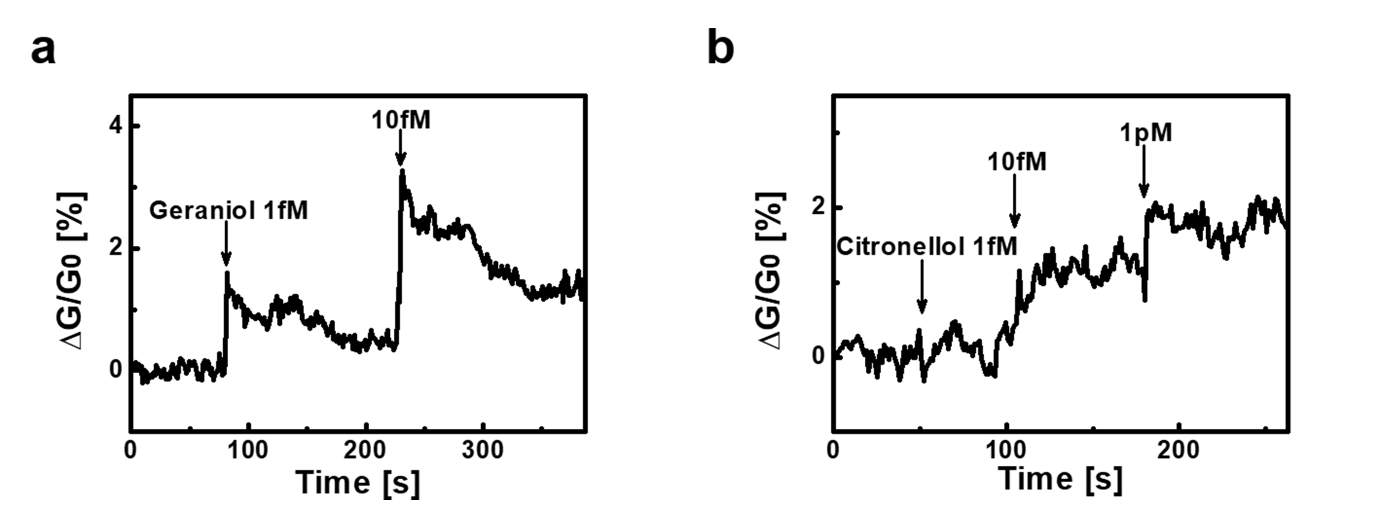


**Figure S3.** (a) Real-time responses of a ND-based bioelectronic nose to various concentrations of geraniol. The bioelectronic nose began to respond to geraniol at the concentration of 1 fM with a signal-to-noise ratio of ~ 4.4. (b) Real-time responses of a ND-based bioelectronic nose to different concentrations of citronellol. The graph shows that the bioelectronic nose responded to citronellol of 10 fM with a signal-to-noise ratio of ~ 5.5.


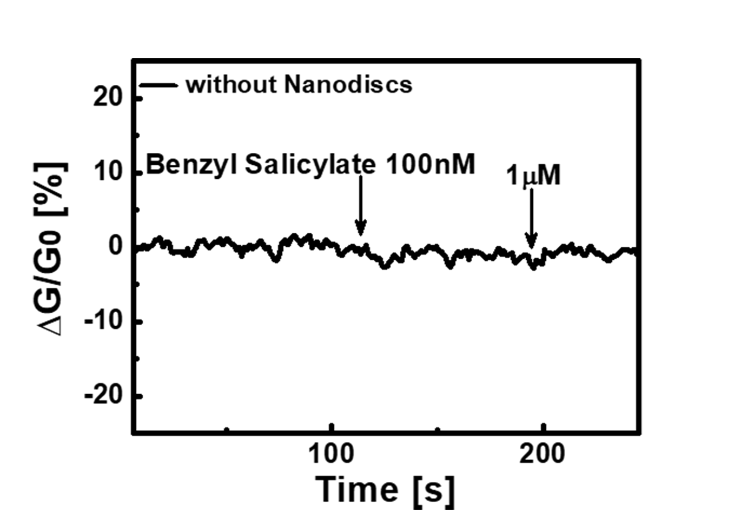


**Figure S4.** Real-time responses of a bare CNT-FET without hOR1A2NDs to varying concentrations of benzyl salicylate. The introduction of benzyl salicylate did not cause the CNT-FET channel conductance change.


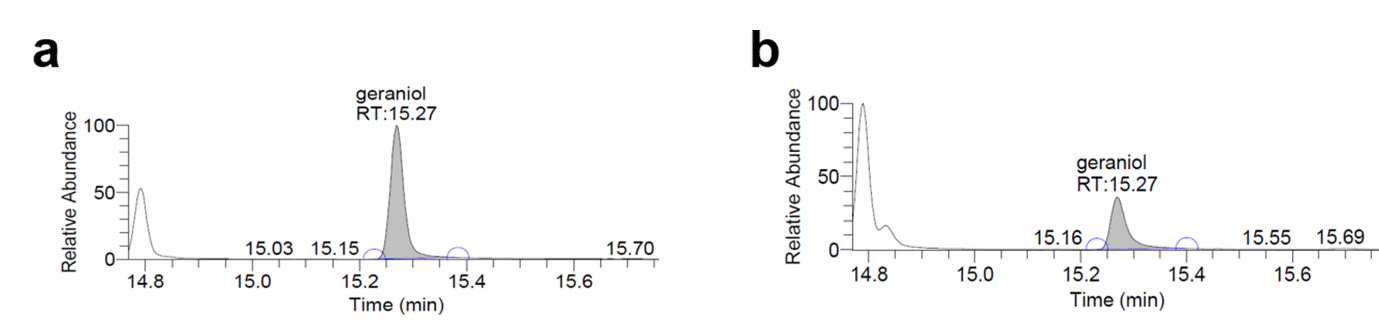


**Figure S5.** (a) GC-MS chromatogram of geraniol of an authentic reference compound. Geraniol of the authentic reference compound has a peak at retention time 15.27 min. (b) GC-MS chromatogram of geraniol in natural rose oil. Geraniol in the natural rose oil has also a peak at retention time 15.27 min. The concentration of geraniol in the undiluted natural rose oil was found to be 9.47 × 10^-4^ M.


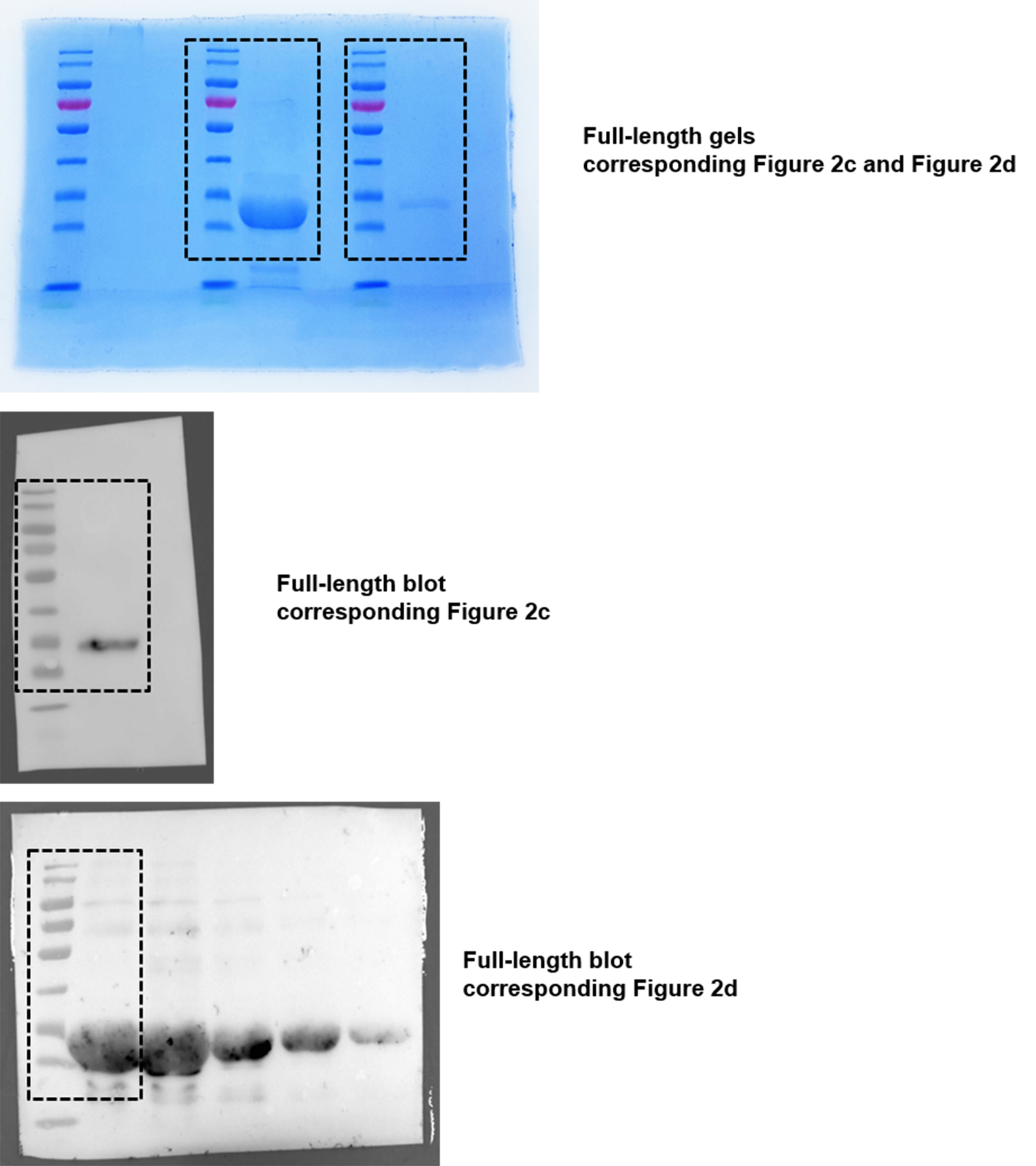


**Figure S6.** (a) Full-length gels and blots corresponding to Figure 2c and Figure 2d. Black dashed lines represent the cropping areas.
